# Evaluating Commercial Loop-Mediated Isothermal Amplification Master Mixes for Enhanced Detection of Foodborne Pathogens

**DOI:** 10.3390/foods13111635

**Published:** 2024-05-24

**Authors:** Ana Costa-Ribeiro, Alexandre Lamas, Alejandro Garrido-Maestu

**Affiliations:** 1 International Iberian Nanotechnology Laboratory, Av. Mestre José Veiga s/n, 4715-330 Braga, Portugal; ana.c.ribeiro@inl.int; 2 Department of Biochemistry, Genetics and Immunology, University of Vigo, 36310 Vigo, Spain; 3 Food Hygiene, Inspection and Control Laboratory (Lhica), Department of Analytical Chemistry, Nutrition and Bromatology, Veterinary School, Campus Terra, University of Santiago de Compostela (USC), 27002 Lugo, Spain; alexandre.lamas@usc.es; 4 Laboratory of Microbiology and Technology of Marine Products (MicroTEC), Instituto de Investigaciones Marinas (IIM), CSIC, Eduardo Cabello, 6, 36208 Vigo, Spain

**Keywords:** loop-mediated isothermal amplification, LAMP, master mix, foodborne pathogens, *L. monocytogenes*, *Salmonella* spp., *E. coli* O157

## Abstract

Loop-mediated isothermal amplification, LAMP, is nowadays the most popular isothermal nucleic acid amplification technique, and as such, several commercial, ready-to-use master mixes have flourished. Unfortunately, independent studies to determine their performance are limited. The current study performed an independent evaluation of the existing ready-to-use commercial LAMP master mixes WarmStart^®^ LAMP Kit, LavaLAMP™ DNA Master Mix, Saphir Bst Turbo GreenMaster, OptiGene Fast Master Mix ISO-004, and SynLAMP Mix. To reduce bias, three different genes, namely *ttr* (*Salmonella* spp.), *rfbE* (*E. coli* O157), and *hly* (*Listeria monocytogenes*), were targeted. The comparison was based on amplification speed, performance with decreasing DNA concentrations, and the effect of five typical LAMP reaction additives (betaine, DMSO, pullulan, TMAC, and GuHCl). Significant differences were observed among the different master mixes. OptiGene provided the fastest amplification and showed less detrimental effects associated with the supplements evaluated. Out of the chemicals tested, pullulan provided the best results in terms of amplification speed. It is noteworthy that the different additives impacted the master mixes differently. Overall, the current study provides insights into the performance of commercial LAMP master mixes, which can be of value for the scientific community to better select appropriate reagents when developing new methods.

## 1. Introduction

In recent years, molecular methods have found a niche in the field of food science. They have been used for food authenticity, the development of rapid methods, microbial detection, and characterization, among others [1,2,3]. Among the wide variety of molecular techniques available nowadays, Polymerase Chain Reaction (PCR) and real-time quantitative PCR (qPCR), with their variants like ERIC-PCR [4], are among the most popular ones; however, these techniques are struggling to cope with the demands of the food industry due to the intensive production systems in place currently. A way to address this need has been to develop different types of miniaturized devices and systems. These can be placed in decentralized setups so that the whole food value chain can be traced, and the analyses can be performed in situ [5,6]. Due to the complexity in terms of equipment of PCR/qPCR, isothermal techniques, such as Loop-mediated isothermal amplification (LAMP), have emerged as suitable alternatives due to their simplicity, rapidity, lower equipment cost, ease of implementation in biosensors, and compatibility with different approaches for easy result interpretation, such as lateral flow or colorimetry [7], making LAMP assays also attractive for fields such as veterinary medicine [8].

Even though LAMP was originally described in 2000 [9], it was not until recently that it attracted the attention of the general scientific community, fundamentally after the COVID-19 pandemic [10,11,12]. Until recently, the number of manufacturers providing the required reagents was limited, and the amount of commercial reagents ready to use, in the format of master mixes, was scarce. In the last 4–5 years, this situation has changed, and now this type of formulation has been reaching the markets. Due to its novelty, independent studies comparing these reagents are very limited, such as the one performed by Domesle et al. [1,13] or Deliveyne et al. [14]. Even though this may seem trivial, the success of any given method relying on this technique is highly dependent on the appropriate selection of reagents. For other techniques, extensive benchmarking of commercial master mixes [15,16], nucleic acid extraction kits [17,18], genetic targets [19,20], and amplification and detection platforms [13,21] has been performed, and they have even been compared to other existing techniques and methods [22,23,24].

Given the current situation, the goal of the present study was to perform a comprehensive comparison among the commercial, ready-to-use master mixes. These were obtained from different suppliers to determine if any of them outperformed the others. To do so, the amplification time was taken into account, as well as the performance of the different products with decreasing concentrations of DNA and the effects that typical LAMP supplements may exert in the amplification. Additionally, to have a better picture, three different LAMP assays were included in the comparison to reduce the potential bias generated by only analyzing the data of one given assay.

## 2. Materials and Methods

### 2.1. Bacterial Strains

The reference strains selected were WDCM 00031 (*Salmonella enterica* serovar Typhimurium), WDCM 00021 (*L. monocytogenes*), acquired from the Spanish Type Culture Collection, and *E. coli* O157:H7 AMC 76, generously provided by the Institute of Applied Microbiology—ASMECRUZ. For all the experiments overnight, fresh cultures were prepared by adding a single colony into Nutrient Broth (NB, Biokar Diagnostics S.A., Allonne, France), and the suspension was incubated at 37 °C overnight.

### 2.2. DNA Extraction and Quantification

A 2 mL aliquot from each fresh culture, prepared as described above, was taken for DNA extraction. The aliquots were spun for 2 min at 16,000× *g*, the supernatant was discarded, and the pellet was resuspended in 1 mL of TE 1X and spun again under the same conditions. After removing the supernatant, the clean pellet was resuspended in 200 µL of Chelex^®^ 100 (6% *w*/*v*, Bio-Rad Laboratories, Inc., Irvine, CA, USA), incubated at 56 °C for 15 min at 1000 rpm in a Thermomixer comfort (Eppendorf AG, Hamburg, Germany), and the bacteria were heat-lysed by further incubating at 99 °C for 10 min at 1400 rpm. Finally, after the lysis step, the aliquots were centrifuged at 4 °C for 2 min at 16,000× *g*. The supernatants were transferred to clean tubes and stored at −20 °C until needed [25].

The nucleic acid concentration was measured in a Qubit™ 4 Fluorometer (Invitrogen™, Carlsbad, CA, USA) using the 1X dsDNA HS Assay Kit (Invitrogen, Carlsbad, CA, USA), and the purity was assessed based on the 260/280 and 260/230 absorbance ratios measured and calculated in a NanoVue™ Plus Spectrophotometer (GE Healthcare Europe GmbH, München, Germany).

### 2.3. LAMP Reaction Setup

A total of five commercial formulations were included in the present study: 1. WarmStart^®^ LAMP Kit (DNA & RNA) (E1700. New England Biolabs, MA, USA); 2. LavaLAMP™ DNA Master Mix (30067-1. Lucigen, Middleton, WI, USA); 3. Saphir Bst Turbo GreenMaster (PCR-393. Jena Bioscience GmbH, Jena, Germany); 4. Fast Master Mix (ISO-004. OptiGene, Horsham, UK); 5. SynLAMP Mix (L011. Synthbiotics, Du-naszentgyörgy, Hungary). From now on, these will be abbreviated and referred to as NEB, Lava, Jena, OG, and Syn, respectively. All the LAMP experiments were performed following the recommendations of the corresponding supplier in regard to optimum primer concentration, temperature, and amplification time, but for better comparison, all the reactions were performed in a final volume of 20 µL with 3 µL of template; see Table 1. ROX (Invitrogen, Carlsbad, CA, USA) was added as a passive reference to all the mixes, and the corresponding free volume was filled with nuclease-free water (New England BioLabs, Inc., Ipswich, MA, USA). All the experiments were conducted in technical triplicates, unless indicated differently, in a QuantStudio™ 5 Real-Time PCR System (Applied Biosystems™, Foster City, CA, USA) and results were analyzed using the respective software QuantStudio™ Design & Analysis version 1.5.1. In all cases, the amplification was performed for 30 min (60 cycles of 30 s with fluorescence acquisition after each cycle) at 65 °C, except for Lava as the manufacturer recommended 68 °C. After amplification, a melt curve analysis was performed from 60 to 95 °C.

To account for variability associated with target/primer sequences, three different genes were targeted in independent reactions to evaluate the performance of the different master mixes, namely *ttr*, *rfbE*, and *hly* for *Salmonella* spp., *E. coli* O157, and *L. monocytogenes*, respectively. The corresponding sequences were taken from reference genomes, namely NC_016856, NZ_CP008957, and NC_003210, and the design of the primers was performed with Primer Explorer V5 (https://primerexplorer.jp/lampv5e/index.html). In Table 2, the specific sequences are provided.

### 2.4. Initial Screening

An initial screening of the different mixes was performed by comparing the time to threshold (*Tt*) of each mix for all three target genes to determine the fastest one.

### 2.5. DNA Concentration Effect

To determine how each of the commercial products performed with different DNA concentrations, ten-fold serial dilutions of the original extracts were performed in nuclease-free water, covering four orders of magnitude to assess the effect of decreasing DNA concentration in the performance of the different LAMP assays.

### 2.6. Supplement Effect

The last part of the comparison consisted of the evaluation of the effect different chemicals had individually on LAMP reactions. These compounds are typically added to enhance sensitivity and/or specificity. In this regard, typical concentrations, previously reported, of the most common supplements were included, namely betaine (0.8 M [27]) and dimethyl sulfoxide (DMSO, 7.5% [28]), as well as others recently reported to improve the results of the other two, like pullulan (1% [29]), Tetramethylammonium Chloride (TMAC, 60 mM [30]) and Guanidine Chloride (GuHCl, 40 mM [31]). The concentration of each supplement was selected based on their typical range of application. To better see the effect of each supplement, the amplification time was extended up to 60 min, and it was decided to use dilution −2 for *ttr* and *rfbE* (0.65 and 0.48 ng/µL, respectively) and dilution −1 for *hly* (0.032 ng/µL). The effect of the different supplements was assessed based on the % of variation in the *Tt* generated in the same reaction without any supplement, as a reference, which was calculated as *Variation % =* 1 − (*Ttsup/Ttref*) ∗ *100*.

### 2.7. Final Evaluation

A matrix was constructed to summarize and evaluate the results obtained from the previous experiments in order to evaluate the different products. A total of seven parameters were considered, with the amplification time, performance with low DNA concentration, and susceptibility to supplements considered key parameters and given 1–5 points, while thermostability, convenience, flexibility, and price were considered “bonus features” and so ranked as 1 or 0. Under “thermostability”, it was evaluated whether the product was stable out of typical refrigeration temperatures. In the category “convenience”, it was evaluated whether the master mix evaluated was completely ready to be used, without any other reagent. When talking about “flexibility”, it was considered whether it could be combined with other chemicals, for instance, different fluorescent dyes. The last category considered within the “bonus features” was the cost per reaction, which was evaluated by attending to the quotations received from the different manufacturers.

### 2.8. Amplicon Sequence Analysis

The whole amplicons generated between the corresponding F3/B3 primers were analyzed with NUPACK [32] and IDT’s OligoAnalyzed (https://eu.idtdna.com/calc/analyzer) to evaluate the presence of secondary structures within the native fragments, as well as %GC.

### 2.9. Data Representation and Statistical Analysis

Graphical representation and data analyses were performed with GraphPad Prism version 8.0.0 for Windows (GraphPad Software, San Diego, CA, USA, www.graphpad.com). The comparison of the *Tt* values obtained was performed by two-way ANOVA with Tukey post-hoc test (*p*-value < 0.05).

## 3. Results

### 3.1. Initial Screening

**Figure 1:**
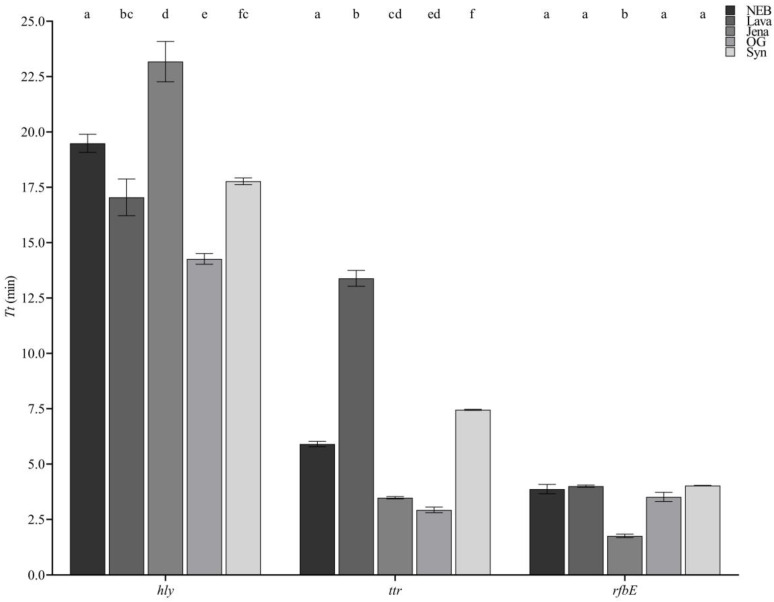
Initial master mix screening reporting the *Tt* of NEB, Lava, Jena, OG, and Syn when amplifying the *hly*, *ttr*, and *rfbE* genes. Within each gene, different letters indicate statistically significant differences.

The genes *ttr*, *hly*, and *rfbE*, typically used for the detection of *Salmonella* spp., *Listeria monocytogenes*, and *E. coli* O157, were targeted in the present study. Statistically significant differences in the *Tt* values obtained with the different master mixes for the different genes were observed. As depicted in Figure 1, the highest number of differences was observed for the amplification of *hly*, where all the mixes, except for Lava vs. Syn, performed differently, with OG achieving the fastest amplification while Jena was the slowest. When targeting *ttr*, again OG resulted in the fastest mix; however, this time, Lava was the slowest. Finally, for the amplification of *rfbE*, Jena was significantly faster than all four other commercial preparations under study.

### 3.2. DNA Concentration Effect

#### 3.2.1. Effect on *hly*

**Figure 2:**
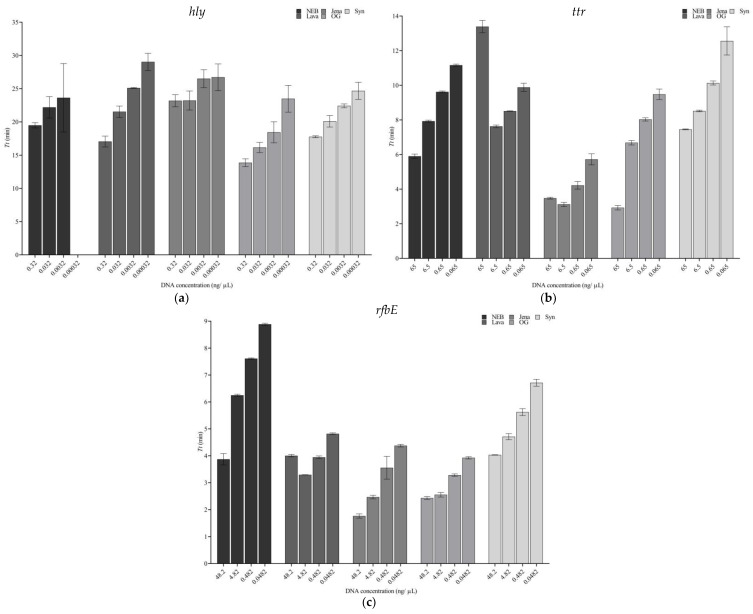
Concentration effect on the different master mixes targeting *hly* (**a**), *ttr* (**b**), and *rfbE* (**c**).

In the four-log dynamic range, evaluated differences were observed for the different targets. When analyzing *hly*, out of the five commercial products, NEB was the one that provided the worst results, as it was not possible to detect the lowest concentration (0.00032 ng/µL), and presented the largest SD in the last concentration amplified (0.0032 ng/µL); see Figure 2a. With the rest of the formulations, in general, an increase in the *Tt* value was observed when decreasing the DNA concentration, also accompanied by an increase in the corresponding SD, and it was noteworthy in the case of Jena, where no differences were observed among the first two dilutions and the last two. The master mix formulated by OG proved to be the fastest one in the concentration range tested.

#### 3.2.2. Effect on *ttr*

When the analysis was focused on *ttr*, it was observed that all the dilutions tested were correctly amplified within the first 15 min with all the mixes. However, Lava presented a significantly higher *Tt* value with the highest DNA concentration, which decreased when analyzing the first dilution; a similar phenomenon was observed with Jena. Like in the case of *hly*, OG happened to be the master mix performing the fastest amplification. These data are depicted in Figure 2b.

#### 3.2.3. Effect on *rfbE*

Finally, when the analysis and comparison were focused on the amplification of the *rfbE* gene, similar results to those of *ttr* were observed. The amplification of the dilutions tested was successfully performed within 10 min, and in line with what was described previously, the highest concentration tested obtained a higher *Tt* than the first dilution; however, the difference was not as high as previously observed for *ttr*. In addition to this, minor differences were observed in the two highest concentrations when analyzed with OG. However, it is important to note that Jena was the fastest one at the highest DNA concentration; see Figure 2c.

### 3.3. LAMP Reaction Supplements Effect

**Figure 3:**
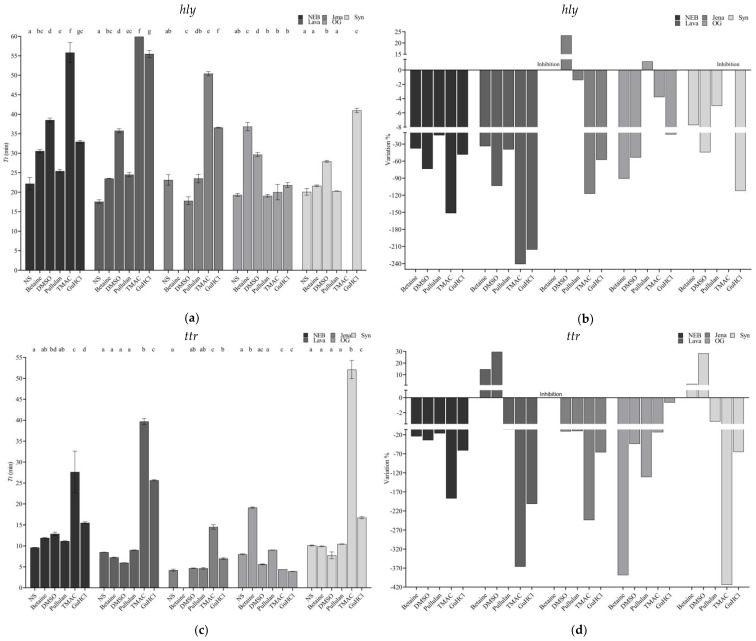

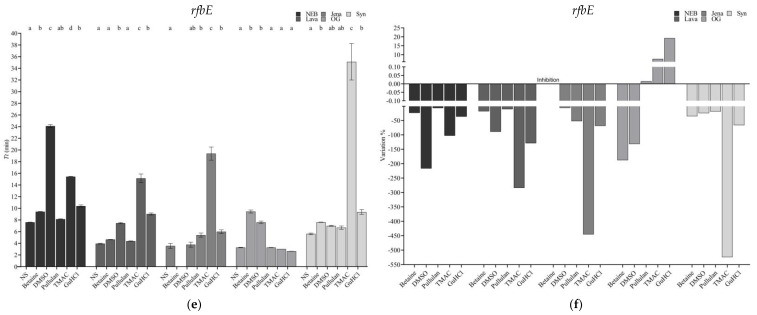
Effect of different supplements on the master mixes compared to native reactions targeting *hly* (**a**); *ttr* (**c**); and *rfbE* (**e**). Percentage variation in the amplification time caused by the supplementation of the reactions targeting *hly* (**b**), *ttr* (**d**), and *rfbE* (**f**). Within each master mix, different letters represent statistically significant differences.

It can be observed in Figure 3a–f that all the supplements exerted some effect on the amplification of all three targets. In general, all tended to increase the *Tt*. Very few combinations of supplement–master mix resulted in reaction acceleration, and this was not consistent among the different genetic targets. The most drastic effects were observed after the addition of betaine to Jena, which caused the complete inhibition of the reaction regardless of the target selected. Similarly, supplementation of Syn with TMAC inhibited the amplification of *hly* and generated a significant delay for *ttr* and *rfbE*. Overall, the supplement that exhibited the most detrimental effect was TMAC, followed by betaine.

When comparing the *Tt* values obtained with each supplement against the native reaction, it was observed that three out of the five agents, namely pullulan, TMAC, and GuHCl, tested did not cause any significant effect on OG when targeting *hly* (Figure 3a,d). These same results were obtained when targeting *rfbE* (Figure 3b,e). When focusing on the amplification of *ttr*, the mixes reporting a higher number of non-significant differences were Lava and Syn; in both cases, the results were obtained when supplementing with betaine, DMSO, and pullulan (Figure 3c,f).

Considering all the supplements and genetic targets, the master mix reporting the higher number of non-significant differences of the *Tt* values with/without supplementation was OG followed by Syn, Lava, Jena, and NEB.

### 3.4. Amplicon Analyses

**Figure 4:**
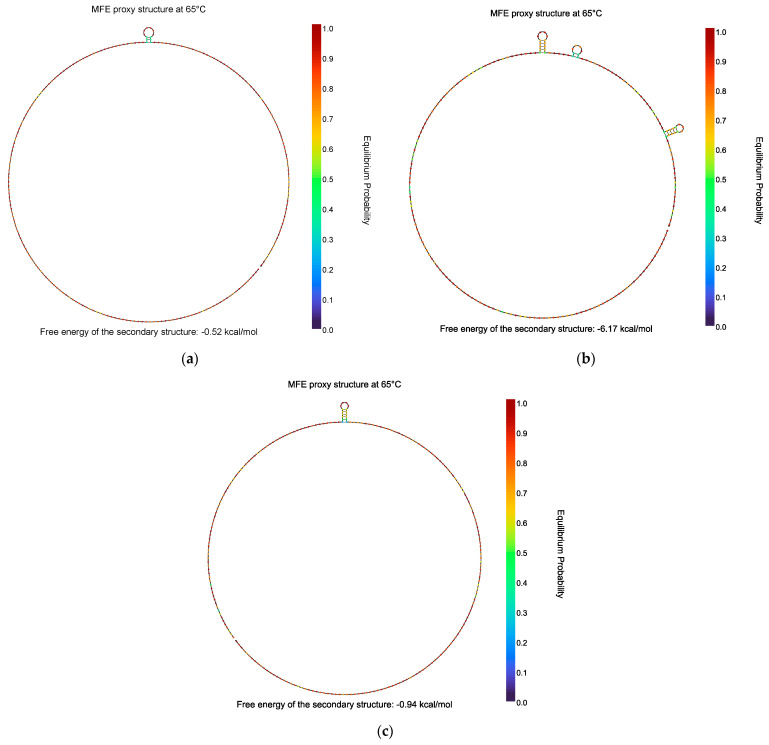
Nupack analysis of the secondary structures of *hly* (**a**), *ttr* (**b**), and *rfbE* (**c**) at 65 °C.

NUPACK analyses were performed to assess the presence of secondary structures that may hinder primer hybridization or amplification. The results obtained did not show any kind of complex secondary structure, which may justify the significant differences observed among the three different genes; see Figure 4a–c. Similarly, the results retrieved from the OligoAnalyzer did not seem to fully justify the significant differences observed among the targets as the fragment size was very similar at ~200 bp, with a similar theoretical Tm of ~70 °C; the major difference would be the %GC of *ttr*, 55.6%, compared to *hly* and *rfbE*, ~36%; however, *ttr* and *rfbE* exhibited similar amplification times. Similarly, the analysis of the secondary structures of each initial amplicon does not seem to justify these differences as the Tm of the theoretical structures are below 50 °C; thus, they should not represent a problem in the running temperature of the reported LAMP experiments, 65–68 °C. These data are summarized in Table 3.

### 3.5. Final Evaluation

The scores given for the key parameters are based on what has been reported so far in terms of speed of amplification, *Tt*, performance with decreasing DNA concentrations, and variations upon supplementation. In regard to the “bonus features”, NEB and Syn were given 1 point as the first was described to be of “warm start” modification, blocking non-desired amplification at room temperature, and the latter was reported to be stable, without significantly losing amplification efficiency after 14 days of storage at 25 °C; as the other three brands did not report anything in this regard, they were given a 0. In the category of “convenience”, Jena and OG, for which only primers and template have to be added, were given 1 point, while NEB, Lava, and Syn require the addition of a fluorescent dye and so scored 0 points. Proceeding to the category of “flexibility”, as NEB, Lava, and Syn require the addition of an external dye, the one provided by the manufacturer may be replaced by a different one if desired, such as SYTO 9 or any other suitable one [33,34], or a different detection strategy can even be used, like turbidity. This approach is not feasible with Jena or with OG, as the dye is already included in the mix. Additionally, in the particular case of OG, to improve fluorescence detection, the mix comes with a pyrophosphatase to degrade the magnesium pyrophosphate [35,36], which may hinder fluorescence detection (OG provides a turbidity reference in its product portfolio); thus, NEB, Lava, and Syn were given a 1 and Jena and OG a 0. Finally, the cost per reaction was also taken into account, setting a cut-off of EUR 2 per reaction, so the most economic reagents, Jena, OG, and Syn were given a 1 while NEB and Lava were given a 0 as they were more expensive (price calculation based on quotations provided by the different manufacturers and/or suppliers). According to the scores obtained by the different products in all seven categories, the best product was OG, followed by Syn, Jena, Lava, and NEB. These results are summarized in Table 4.

The overall evaluation of the results obtained in seven categories, three key master mix features, and four bonus features, evaluated for all the products when analyzing three different genetic targets indicated that the best-performing product was OG, which obtained 17 out of the 19 points, followed by Syn with 13 which was very closely followed by Jena with 12, and the worse results were obtained with Lava and NEB which were given 9 and 6 points respectively, see Table 4.

## 4. Discussion

LAMP has become a very popular technique in recent years, particularly for the detection of different types of microbial pathogens, as well as for the development of miniaturized devices to be used in decentralized setups [7,37,38]. Until recently, a limited number of suppliers were providing the needed reagents to perform it, and in most cases as individual reagents for the researcher to build up the final reaction mix. However, due to its popularity increase among the scientific community, the number of commercial, ready-to-use LAMP master mixes has increased.

In the current study, it was decided to perform an independent evaluation of five suppliers; to the best of our knowledge, this is the largest study performed so far to determine which commercial formulation performs better. To avoid bias related to target/primer variability, and to better assess the performance of all the commercial products, three different genetic targets were tested. More specifically, the *ttr* gene, which encodes for the tetrathionate reductase, was targeted for the detection of *Salmonella* spp. as tetrathionate respiration should be genetically stable within the genus [39,40]; the gene *hly*, encoding for the listeriolysin O, was chosen for the detection of *L. monocytogenes*, as it has been reported to be relatively well conserved in the species [41,42]; last, the gene *rfbE* was selected for the specific detection of *E. coli* O157 as this gene is the one encoding for the “O” antigen of *E. coli*, and as such, it is highly specific for this serogroup [43,44]. The *Tt* values obtained were directly compared. The evaluation included their performance with decreasing template DNA concentrations, and with different types of supplements typically used to enhance LAMP reactions, either by increasing the speed or specificity of such reactions.

In relation to the *Tt* values obtained with the highest DNA concentrations for each bacterial pathogen, the fastest master mix was OG, which achieved the fastest amplification for *hly* and *ttr*; similarly, Jena was the fastest for the amplification of *ttr* and *rfbE;* however, as it was the worst performing with *hly*, OG was considered superior.

In terms of performance with decreasing DNA concentrations, a similar trend was overall observed with the different formulations as described above. However, it was noted that NEB seemed to perform worse than the rest with *hly*, where it failed to amplify the lowest concentration, 0.00032 ng/µL. Additionally, with 0.0032 ng/µL, it presented the highest SD of all the commercial products. This type of tendency was not observed with the other genetic targets, *ttr* and *rfbE*; however, this may be due to a higher DNA concentration even in the lowest concentration tested. Other than this, it was remarkable that some mixes, namely Lava and Jena, seemed to be more susceptible to an excess of template as the *Tt* values obtained with the highest concentrations of *ttr* and *rfbE* were higher than those of the first dilution, while the expected tendency of increased *Tt* was observed from dilution −1 onwards, as depicted in Figure 2a–c. This observation may be related to excess DNA as has also been reported in PCR/qPCR [45,46]. Overall, when targeting *hly*, OG obtained the lowest *Tt* values, Jena performed the best targeting *ttr* and both mixes returned similar results for *rfbE*.

Due to the relative novelty of LAMP, several studies have been published attempting to improve its performance, and they were mainly focused on reducing unspecific amplifications by supplementing the reactions with different types of chemicals [47]. Moving forward in the comparison, it was decided to evaluate the effect of five typical LAMP supplements on each one of the mixes. To do so, the most concentrated DNA extract was not used to avoid any potential mimicking effect this would have, and diluted extracts were selected. It was clear that these chemicals exerted different effects on the different mixes. As a general observation, TMAC was the reagent causing the most detrimental effect as it negatively impacted the amplification in 8 out of the 15 combinations of the master mix–genetic target tested. It was important to note that it very significantly impacted the amplification of the three targets when using Syn, for which the supplier advised against supplementing with GuHCl (https://synthbioenzymes.com/wp-content/uploads/2022/07/Manual-SynLAMP.pdf), which also delayed the amplification, but they did not mention TMAC, which actually inhibited the amplification of *hly*. TMAC was surprisingly followed by betaine as the supplement that most significantly increased the *Tt* values; however, in this last case, it is important to clarify that this chemical only impacted OG and Jena, but in the case of the last one, its addition caused a complete reaction inhibition, while with OG, it simply delayed the amplification. These observations were constant among the different genetic targets. The fact that betaine, despite being of generalized use, generates amplification delays was already reported by Ma et al. [48]. In this part of the evaluation, OG was considered the best formulation as when comparing the Tt values obtained without supplement vs. supplement, in all three genes, eight of the combinations did not significantly impact the *Tt* (see Figure 3a,c,e), thus indicating a robust performance, meaning that different types of chemicals may be used to improve its specificity, if needed, without impacting the amplification time. In this regard, the supplements that provided the best results were pullulan, TMAC, and GuHCl.

It is worth mentioning that in the comparison against the native reaction, the supplement that least impacted the *Tt* was pullulan, in agreement with what was previously reported by Gao et al. [29], who also demonstrated its compatibility with other isothermal techniques such as CPA and RCA [49]. None of the supplements accelerated NEB for any of the targets, while betaine and DMSO, particularly the latter, did reduce the amplification time of Lava and Syn when targeting *ttr*, but not the other two targets, which may be related to a decrease in the melting as this target was determined to have a higher %GC; see Table 3 [28,47,50,51,52]. Jena was enhanced by the addition of DMSO when targeting *hly*, and OG with pullulan, which also enhanced the amplification speed targeting *rfbE* with this mix even though less than TMAC and GuHCl as shown in Figure 3b,d,f. As a final note in regard to the effect of the chemicals tested, the authors would like to highlight the fact that all the master mixes are proprietary from different commercial suppliers and as such, a full disclosure of the components of each one has not been provided, and so detrimental effects of a given chemical due to incompatibility with a certain formulation may be expected; this may explain why a typical reagent such as betaine generated a complete reaction inhibition when added to Jena, as already highlighted by Ma et al. and Foo et al. [48,51].

A sequence analysis of the fragments amplified by the different targets was performed to attempt to elucidate if this could explain the differences observed in amplification speed. Unfortunately, none of the analyses shed any light in this regard as one may expect that initial hairpins could reduce the amplification rate, but no such structures were theoretically expected at the amplification temperatures set. Similarly, a high % of GC could also delay the amplification, but *ttr*, which had a significantly higher %GC, was one of the fastest assays even when compared at even concentrations with *hly* and *rfbE*. Additionally, these two targets presented a similar %GC but their *Tt* values, when similar concentrations were compared, were significantly different.

Taken together, our results support, and add to, those of Domesle et al., who already reported the better performance of OG over NEB [13], which are now demonstrated to be better than the other three brands in terms of amplification speed and robustness in the presence of enhancing supplements. These results indicate that OG is, on average, the best option for novel assay development; however, it cannot be ruled out that for specific applications, other commercial products may provide better results. Even though this is the case, it is important to keep in mind that the comparison was performed with the recommended conditions provided by the different manufacturers; thus, it is possible that after assay optimization, a specific brand may outperform the others. Similarly, one single concentration was tested for each supplement, so the performance of a specific brand may change under conditions different from those reported herein.

## 5. Conclusions

In the present study, a comprehensive evaluation and comparison, of five different commercial, ready-to-use master mix formulations for LAMP reactions were performed. To this end, five commercial reagents were included in this study, and their evaluation was based on their amplification speed, performance with decreasing DNA concentrations, and behavior in the presence of five different LAMP reaction supplements when targeting three different bacterial genes. From the results obtained, it can be concluded that OG’s Fast Master Mix ISO-004 is the best-performing product.

## Figures and Tables

**Table 1:** Summary of LAMP conditions recommended for each master mix.

	NEB	Lava	Jena	OG	Syn
FIP/BIP	1600	1600	1600	800	1600
F3/B3	200	200	200	200	200
LF/LB	400	400	400	400	400
Temperature (°C)	65	68	65	65	65
Time (min)	30	30	30–60	30	30
Remarkable feature	Warm start and UDG	High A amplification temperature	-	Speed	Stability at room temperature

The concentrations reported are in nM and correspond to both primers. FIP: Forward Inner Primer. BIP: Backward Inner Primer. F3: forward outer primer. B3: backward reverse primer. LF: forward Loop primer. LB: backward Loop primer. UDG: Uracil DNA Glycosylase. All the reactions were performed in a final volume of 20 µL with 3 µL of template.

**Table 2:** Primers.

Gene	Primer	Sequence 5′-> 3′	Reference
*ttr*	ttr-FIP	GCA TCA GCC AAC ATA GCG CCA *tttt* CTA CGC CAT CCG TTA TCA CA	This study
	ttr-BIP	TCA GGT ACA AAC CGT CCC CAA G *tttt* CAT CCG TTC CGC CTG GTA	
	ttr-F3	ACA CTG CTG TTC TGT AGC CT	
	ttr-B3	AGG TGC CGA GAA TAG CCA	
	ttr-LF	CCA GCA GGA CGC GTC TT	
	ttr-LB	CGC GCA ATT TAA CCC TTA CTC G	
*rfbE*	rfbE-FIP	GGC CTT TAA AAT GTA AAC AAC GGT C *tttt* ACT ACA GGT GAA GGT GGA	This study
	rfbE-BIP	TAG CTG TAC ATA GGC AAT ATT GGC *tttt* AAT CCT ATA GCA GCG CAG A	
	rfbE-F3	TGT GGG AAC ATT TGG AGA T	
	rfbE-B3	CAT CAG CTT GTT CTA ACT GG	
	rfbE-LF	TTT GTC ATT CGT GAC AAC CAT	
	rfbE-LB	AGG CTA CAA TTA TAG GAT GAC AAA T	
*hly*	hly-FIP	TGA ACA ATT TCG TTA CCT TCA GGA T *tttt* TCG ATC ACT CTG GAG GAT AC	[26]
	hly-BIP	GGA GCG AAA ACA ATA AAA GCA AGC T *tttt* GCG TAA ACA TTA ATA TTT CTC GC	
	hly-F3	TTC AAA AGC TTA TAC AGA TGG AA	
	hly-B3	AAG CTA AAC CAG TGC ATT C	
	hly-LF	CAT CCC AAG AAA TGT TGA ATT GAG C	
	hly-LB	TCG TCC ATC TAT TTG CCA GGT A	

The “-*tttt*-” in all FIP/BIP primers is a linker between the corresponding F2-F1c and B2-B1c.

**Table 3:** Summary information of the native amplicon of each target.

Target	Length (bp)	% GC	Melting Temperature (°C) ^1^	Tm Highest Hairpin (°C)
*hly*	236	35.6	73.1	36.8
*ttr*	205	55.6	69.6	47.2
*rfbE*	236	36.9	72.4	38.1

^1^ Tm calculated with Geneious for *hly*, *ttr*, and *rfbE* was 81.1, 88.9, and 81.7 °C, respectively.

**Table 4:** Master mix evaluation summary.

		Key Features		Bonus Features				
	*Tt*	DNA Concentration	Supplements	Thermostability *	Convenience	Flexibility	Price	Total
NEB	2	1	1	1	0	1	0	6
Lava	*3*	2	3	0	0	1	0	9
Jena	*4*	4	2	0	1	0	1	12
**OG**	**5**	**5**	**5**	**0**	**1**	**0**	**1**	**17**
Syn	*3*	3	4	1	0	1	1	13

* This is a feature claimed by the manufacturer and as such has not been experimentally confirmed in the current study. *Tt*: time to threshold, fastest master mix. DNA concentration: performance with low DNA concentration. Supplements: susceptibility, particularly detrimental, of the master mix to different LAMP supplements. Convenience: need to add extra compounds or ready to use. Flexibility: can the reaction be adapted for different applications, e.g., different detection chemistry? Price: cost per reaction higher or lower than EUR 2. The master mix providing the best results is highlighted in bold letters.

## Data Availability

The original contributions presented in the study are included in the article, further inquiries can be directed to the corresponding author.
